# Rationale and design of a large trial of perioperative ketamine for prevention of chronic post-surgical pain

**DOI:** 10.1186/s13063-024-08672-y

**Published:** 2024-12-19

**Authors:** Philip J. Peyton, Sabine Braat, Anurika De Silva, David Story, Lisbeth Evered, Paul S. Myles, Matthew Chan, Stephan Schug, Malcolm Hogg, Alex Holmes, Sofia Sidiropoulos, Kate Leslie

**Affiliations:** 1 Department of Critical Care, Melbourne Medicine School, Faculty of Medicine, Dentistry and Health Sciences, University of Melbourne, Melbourne, VIC Australia; 2 Department of Anaesthesia, Austin Health, Studley Rd., Heidelberg, VIC 3084 Australia; 3 Department of Anaesthesia & Pain Management, Royal Melbourne Hospital, Parkville, VIC Australia; 4 Centre for Epidemiology and Biostatistics, Melbourne School of Population and Global Health, University of Melbourne, Melbourne, VIC Australia; 5 Methods & Implementation Support for Clinical and Health (MISCH) research Hub, Faculty of Medicine, Dentistry and Health Sciences, University of Melbourne, Melbourne, VIC Australia; 6 School of Translational Medicine, Faculty of Medicine, Nursing and Health Sciences, Monash University, Melbourne, VIC Australia; 7 Department of Anaesthesia and Intensive Care, The Chinese University of Hong Kong, Hong Kong, People’s Republic of China; 8 Department of Anesthesiology, Weill Cornell Medicine, New York, NY USA; 9 Department of Anaesthesiology, University of Western Australia, Perth, Australia; 10 Department of Anaesthesia, St Vincent’s Hospital, Melbourne, VIC Australia; 11 Department of Anaesthesiology and Perioperative Medicine, Alfred Health, Melbourne, VIC Australia

## Abstract

**Background:**

Chronic post-surgical pain (CPSP) is recognised as one of the most common and debilitating complications of major surgery. Progression from acute to chronic pain after surgery involves sensitisation of central nervous system pathways with the N-methyl-D-aspartate (NMDA) receptor having a central role. Ketamine is a potent, non-selective NMDA antagonist commonly used for management of acute postoperative pain. Inconsistent but largely supportive evidence from small trials of a preventative effect of perioperative ketamine on CPSP risk suggests that a confirmative large trial is needed.

**Methods:**

The ROCKet (*R*eduction *O*f *C*hronic Post-surgical Pain with *Ket*amine) Trial is a multicentre, double-blind, placebo-controlled, individually randomised superiority trial conducted in 36 hospitals across Australia, New Zealand, and Hong Kong. The trial aims to recruit 4884 patients undergoing abdominal, thoracic, or major orthopaedic surgery. Eligible participants are randomised equally to perioperative intravenous ketamine or placebo for up to 72 h. Incidence of pain in the area of the index surgery is measured by structured telephone interview at 3 months (primary trial endpoint) and 12 months. Pain severity, nature, and associated psychological and quality of life indices are measured using the modified Brief Pain Inventory short form, Neuropathic Pain Questionnaire, Kessler K-10 Psychological Distress Scale, Pain Catastrophising Scale, EQ-5D-3L, and measures of healthcare utilisation and costs. The trial is being conducted by the Department of Critical Care, University of Melbourne, and the Australian and New Zealand College of Anaesthetists Clinical Trials Network. The trial is funded by the Australian National Health and Medical Research Council.

**Discussion:**

The ROCKet trial will clarify the effectiveness of ketamine in primary prevention of CPSP. In addition, it will provide high-quality, prospective data on the epidemiology of CPSP which will better inform further research into prevention and management of CPSP.

**Trial registration:**

Australian New Zealand Clinical Trials Registry (ACTRN12617001619336) on the date of 12/11/2017.

## Background

Chronic post-surgical pain (CPSP) is recognised as one of the most common and debilitating complications of major surgery [1]. A number of surgical and psychosocial factors are associated with an increased likelihood of developing CPSP including severity of early (acute) postoperative pain, increased duration of surgery, open (as opposed to laparoscopic) surgical approach, intraoperative nerve damage, preoperative anxiety and fear, introversion, previous persistent pain, and mental health conditions [2–4].

Investigators studying the epidemiology of CPSP have highlighted that ‘an alarming proportion of patients…develop chronic postsurgical pain…’ and ‘… even the lowest incidences are unacceptably high’ [2, 3]. A review of CPSP found ‘one-year incidence to be highly variable and surgery specific, ranging from a low of approximately 10–15% following modified radical mastectomy…to a high of 61–70% for thoracotomy…and amputation’ [3]. These data are disturbing but are confirmed by a Norwegian national epidemiological study, which found an incidence of 40% at least 3 months post-surgery, and by prospective follow-up data from the large international ENIGMA (Evaluation of Nitrous oxide In the Gas Mixture in Anaesthesia) and ENIGMA II trials, which found an overall incidence of CPSP of 11–12% at 1 to 4 years after a broad range of major surgery [5–7]. There is relatively little data on the natural history of CPSP, and it is likely that the variation in incidence reported by studies reflects heterogeneity in surgical type and the methodology and timing of data collection which typically ranges between 3 months and 1 year post-surgery. A taskforce of the International Association for the Study of Pain (IASP) has recommended that chronic pain be defined as pain persisting for at least 3 months, as part of a new World Health Organisation (WHO) ICD-11 definition of chronic pain as a discrete disease state [8]. This definition has been extended to include CPSP and post-traumatic pain [9].

Progression from acute to chronic pain after surgery and trauma involves sensitisation of central nervous system pathways, particularly at the level of the spinal cord, neuroinflammation, and spinal cord ‘wind-up’, with the N-methyl-D-aspartate (NMDA) receptor having a central role in this process [10–12]. Ketamine is a potent, non-selective NMDA antagonist which is commonly used as a second- or third-line agent for management of acute postoperative pain, because of its unique analgesic properties [13]. Ketamine administration can lead to psychomimetic or dissociative side effects in around 5% of surgical patients, including vivid dreams, hallucinations, and agitation, which limit dosage in a small proportion of patients in the postoperative setting [13].

The potential effect of ketamine on the development of CPSP is unclear. A Cochrane review examined the effect on CPSP incidence of several perioperative pharmacological interventions including gabapentinoids, membrane stabilisers, steroids, non-steroidal anti-inflammatories (NSAIDs), and NMDA receptor antagonists including nitrous oxide and ketamine. Across eight heterogeneous small studies totalling 914 patients, the authors found evidence of a potential benefit from intravenous (IV) ketamine, with an odds ratio (OR) versus placebo of 0.50 (95% confidence interval [CI] 0.33–0.76, *p* = 0.001) [14]. They cautioned that large trial data were lacking and needed. A more recent meta-analysis has reinforced this conclusion [15]. An extensive James Lind Alliance survey of 2000 health practitioners, patients, and public stakeholders in the UK nominated prevention of CPSP as a leading research priority for anaesthesia and perioperative medicine [16].

The ROCKet (*R*eduction *O*f *C*hronic post-surgical pain with *Ket*amine) trial is a large, multicentre, double-blind, parallel group, placebo-controlled, individually randomised superiority trial of the effect of perioperative IV ketamine on the incidence and severity of CPSP, measured at 3 months and 12 months following elective abdominal, thoracic, or major orthopaedic surgery. The trial is being conducted by the Australian and New Zealand College of Anaesthetists Clinical Trials Network (ANZCA CTN).

## Methods/design

### Objectives

The primary objective is to determine if IV ketamine, given immediately prior to and for up to 72 h following surgical incision, reduces the incidence of CPSP at 3 months after surgery, compared to placebo.

Secondary objectives include measuring the effect of perioperative ketamine on indices of acute postoperative pain severity, side effects, and quality of recovery. Ketamine effect on CPSP incidence at 12 months is also being measured. Measures of CPSP severity and neuropathic characteristics, and patient wellbeing and psychological distress relative to preoperative baselines, are assessed at 3 and 12 months post-surgery, including their relationship to trial treatment allocation. The trial protocol is described according to the SPIRIT (Standard Protocol Items Recommendations for Interventional Trials) reporting guidelines (see SPIRIT Checklist in the supplementary material) [17]. Table 1 outlines the protocol using the recommended WHO trial registration data set.

**Table 1:** WHO trial registration data set

Data category	Information
Primary registry and trial identifying number	Australian and New Zealand Clinical Trials Registry: ACTRN12617001619336
Date of registration in primary registry	11 December 2017
Source(s) of monetary or material support	NHMRC Australia: Project Grant (GNT1120848, 2017) Clinical Trials and Cohort Studies Grant (GNT2023989, 2022); Research Grants Council Hong Kong: (Grant 14112718)
Primary sponsor	University of Melbourne, Victoria, Australia
Contact for public queries	Prof. Philip Peyton phil.peyton@austin.org.au
Contact for scientific queries	Prof. Philip Peyton phil.peyton@austin.org.au
Public title	ROCKet Trial
Scientific title	*R*eduction *O*f *C*hronic Post-surgical Pain with *Ket*amine (ROCKet) Trial
Countries of recruitment	Australia, New Zealand, Hong Kong
Health condition or problem studied	Chronic post-surgical pain (CPSP) at 3 months (ICD-11 definition) after index surgery
Intervention	
Active comparator:	IV ketamine commenced prior to surgical incision and continued for up to 72 h or until hospital discharge
Placebo comparator:	Matched IV saline placebo
Key inclusion criteria (see Table 2)	Consenting adult patients (≥ 18 years), ASA 1–3, undergoing elective or expedited surgery and anaesthesia with planned postoperative opioid analgesia and hospital stay of at least one postoperative night, for any one of the following: I. Abdominal surgery involving an expected skin incision at least 8cm in length, and including open inguinal herniorrhaphy II. Non-cardiac thoracic surgery, including mastectomy and breast reconstruction surgery and including all video-assisted thoracoscopic surgery (VATS) III. Hip, knee and shoulder joint arthroplasty and spinal surgery involving an expected skin incision at least 8 cm
Key exclusion criteria (see Table 2)	Age > 85 years, pregnancy, BMI > 45 kg/m^2^ or weight over 130 kg, ASA 4 or 5 Planned use of intra- or post-operative continuous IV lignocaine infusion Contraindications to IV ketamine administration
Study type	Interventional, phase III trial
	Primary purpose: prevention
	Allocation: randomised 1:1, stratified by (i) hospital, (ii) preoperative pain at surgical site Intervention model: parallel assignment Masking: double blind
Date of first enrolment	19 December 2017
Target sample size	4884
Recruitment status	Recruiting
Primary outcome	CPSP incidence at 3 months after index surgery
Key secondary outcomes	*Within 3 months after index surgery*: Acute postoperative pain severity (opioid consumption oral morphine equivalents, and mean, minimum and maximum NRS pain scores at rest and activity) on days 1–3 post-surgery (or until hospital discharge) Quality of recovery on day 1 using the QoR-15 Length of hospital stay defined as time from surgery start to hospital discharge Incidence of adverse/side effects related to the study drug during index hospital admission
	Severity of CPSP at 3 months after index surgery (mBPI) and neuropathic pain incidence (NPQ) Wellbeing and psychological distress at 3 months post-surgery (mBPI, EQ-5D-3L and K-10)
	*Within 12 months after index surgery*: CPSP at 12 months after index surgery; severity of CPSP at 12 months after index surgery (mBPI) and neuropathic pain incidence (NPQ) Wellbeing and psychological distress at 3 months post-surgery (mBPI, EQ-5D-3L and K-10)

*WHO* World Health Organization, *ICD-11* International Classification of Diseases 11th Revision, *NHMRC* National Health and Medical Research Council, *IV *Intravenous, *CPSP *Chronic post-surgical pain, *ASA *American Society of Anesthesiologists physical status classification, *BMI* Body mass index, *mBPI *Modified Brief Pain Inventory, *NRS* Numerical Rating Scale, *NPQ* Neuropathic Pain Questionnaire, *EQ-5D-3L* EQ-5D-3L questionnaire, *K-10* Kessler K-10 questionnaire

The trial protocol is designed to make a large multicentre trial of a perioperative intervention with a delayed primary outcome feasible, using a distance model of postoperative telephone follow-up for primary endpoint data collection that was successfully used in two previous large multicentre ANZCA CTN trials (ENIGMA and ENIGMA II) [6, 7]. The trial protocol was piloted in the ROCKet Pilot trial, conducted in three hospitals in Victoria, Australia, recruiting 80 patients undergoing abdominal, thoracic, or breast surgery. CPSP was measured at 6 months post-surgery in this pilot trial [18].

Identical data on CPSP at both 3 months and 12 months post-surgery has been collected from the trial outset. Originally, CPSP at 12 months post-surgery was chosen as the primary trial endpoint, to be consistent with data on CPSP incidence collected using similar methodology at 12 months after surgery from 3000 patients enrolled in the ENIGMA II trial [6, 19]. These data were used to estimate the expected CPSP incidence and data attrition rates for sample size calculation for the ROCKet trial. However, after ICD-11 was formally implemented by the WHO in 2022, the trial steering committee, who remained fully blinded to trial treatment allocation at all times, decided to change the timing of the ROCKet trial primary endpoint to 3 months post-surgery, with treatment effect at 12 months post-surgery becoming a secondary endpoint.

### Ethics, sponsorship, and trial registration

Approval was granted by the Austin Health Human Research Ethics Committee in 2017 (HREC17Austin161), and the trial was registered with the Australian New Zealand Clinical Trials Registry (ACTRN12617001619336, 11 December 2017). Trial sponsorship is provided by the University of Melbourne (Parkville 3050, Victoria, Australia).

### Governance

The design and conduct of the trial are the responsibility of the trial steering committee, consisting of the chief investigators, trial statistician, and trial manager. Monitoring of trial protocol delivery and data quality is managed by the trial operations committee. A data and safety monitoring committee (DSMC), consisting of 5 independent experts in clinical trials and an independent statistician, review unblinded data on safety endpoints and adverse events, monitor withdrawals, and review ethical conduct of the trial. In addition, they advised on the pre-planned interim analysis. A single pre-planned interim analysis by the DSMC was scheduled that allowed stopping early for superiority, at the availability of 12-month follow-up CPSP data from the first 1500 recruited patients (expected at roughly around the mid-point of overall trial recruitment).

### Population

The inclusion criteria represent consenting adult patients (18 years or more) presenting for elective or expedited surgery under anaesthesia for a variety of major surgeries (Table 2) associated with CPSP. The exclusion criteria cover a range of relative or absolute contraindications to ketamine administration or to participation in the trial (Table 2).

**Table 2:** ROCKet trial recruitment inclusion and exclusion criteria

**Inclusion criteria**
Written informed consent
Age ≥ 18 years
ASA physical status 1–3
Elective or expedited surgery and anaesthesia for any one of the following:
I. Abdominal surgery involving an expected skin incision at least 8cm in length, and including open inguinal herniorrhaphy
II. Non-cardiac thoracic surgery, including mastectomy and breast reconstruction surgery and including all video-assisted thoracoscopic surgery
III. Hip, knee, and shoulder joint arthroplasty and spinal surgery involving an expected skin incision at least 8 cm
Plan for postoperative opioid analgesia
Plan for hospital stay of at least one postoperative night
Expected to be alive at 12 months after index surgery
**Exclusion criteria**
Age > 85 years
Unable to provide written informed consent
Pregnancy or lactation
BMI > 45 kg/m^2^ and weight over 130 kg
ASA physical status 4–5
Planned use of intra or postoperative continuous IV lignocaine infusion
Uncontrolled hypertension (SBP > 180 mmHg) on admission
Poorly controlled atrial fibrillation (ventricular response rate > 120/min) on admission
Uncontrolled heart failure
Intracranial surgery or raised intracranial pressure
History of haemorrhagic stroke
Severe impairment of liver function
Previous adverse reaction to ketamine
Documented complex regional pain syndrome
History of epilepsy or convulsions
History of psychosis or of illicit drug use or known illegal activities
Previously randomised to the ROCKet trial

*ASA *American Society of Anesthesiologists, *BMI *Body mass index, *SBP *Systolic blood pressure

The trial is being conducted in metropolitan and regional acute care hospitals in Australia, New Zealand, and Hong Kong. Participating sites have access to an acute pain service within their hospital and the equipment available to administer trial drug intraoperatively and postoperatively. Patients may be co-enrolled in other studies where there is no conflict in trial design, intervention, or outcome determination with the ROCKet trial. The trial is managed by the trial coordinating centre at the Department of Critical Care, the University of Melbourne, Victoria, Australia.

### Recruitment

Patients are screened for eligibility preoperatively via surgical booking lists and approached by research staff by telephone or at preadmission surgical or anaesthesia outpatient clinics or on the surgical ward for preoperative inpatients. The patient information and consent form is provided along with a face-to-face discussion with eligible patients and carers or family, to answer any questions about the intervention and potential risks and benefits and to allow written, informed consent to be given by the patient. A model consent form is provided as a supplementary material.

### Randomisation and treatment allocation

Following informed consent and liaison with clinical staff identified as responsible for perioperative care of the patient, randomisation is achieved by research staff preoperatively on the day of surgery using a password-protected web-based system. Randomisation is stratified by (a) hospital and (b) presence or absence of preoperative pain in the area of the surgery (Numerical Rating Scale [NRS] score for average pain in last 24 h of ≥ 3/10). Patients are randomly assigned with a 1:1 allocation to either the intervention arm (ketamine intra- and postoperatively) or the control arm (placebo intra- and postoperatively). The randomisation code was computer-generated using randomly permuted blocks by an independent statistician from the University of Melbourne.

### Blinding

Trial participants, research, hospital pharmacy, medical and nursing staff, data collectors, trial statistician, and Steering, Operations and Endpoint Adjudication Committee (EAC) members are all blinded to trial group assignment until after completion of primary endpoint data collection and database finalisation and locking.

### Intervention

The trial solution (racemic ketamine or normal saline placebo) is formulated and packaged by the manufacturer in compliance with good manufacturing practice (GMP) manufacturing and labelling standards and the Pharmaceutical Inspection Co-operation Scheme (PICS) code of medicinal manufacturing, with active drug and placebo solutions presented in identical form in individual numerically coded vials. Allocation of the correct vials to a randomised patient is controlled by the drug management module within the trial electronic database and communicated to research staff via the web-based portal after randomisation.

Patients randomised to the intervention arm are given an intraoperative loading dose of 0.5 mg/kg (capped at 100 kg body weight) ketamine, after induction of anaesthesia and prior to surgical incision. This is immediately followed by an infusion of 0.125 mg/kg/h which runs until wound closure. This relatively low infusion rate sits within the dose range where inhibition by ketamine of opioid induced hyperalgesia has been demonstrated, which is mediated by the NMDA receptor and is commonly seen in opioid use for chronic pain [20, 21]. This initial dose was chosen as the most likely to generate any potential mechanistic benefits of ketamine in inhibiting central nervous system sensitisation and chronic pain establishment, while minimising side effects. The infusion is recommenced in the post anaesthesia care unit (PACU) at the same rate and continued for up to 72 h while the patient is receiving postoperative opioid analgesia and has ongoing IV access. The placebo arm receives a loading and infusion of the placebo solution at an identical volume and infusion rate.

The pragmatic trial protocol is designed to be compatible with modern principles of early mobilisation and enhanced recovery after surgery, with cessation of the infusion where IV access and/or opioid analgesia is ceased or the patient is being prepared for hospital discharge. The infusion rate can be adjusted by escalation in response to severe or refractory acute postoperative pain (increase to 0.25 mg/kg/h) or reduction if problematic ketamine induced side effects are suspected (reduction to 0.0625 mg/kg/h with cessation after a further 6 h if clinically indicated). If, due to ongoing refractory acute postoperative pain despite dose escalation (e.g. Numerical Rating Scale (NRS) pain scores > 5/10 at rest with functional impairment of postoperative mobilisation), an open label ketamine infusion is considered necessary by the treating clinicians, this can be commenced and the trial infusion ceased, and the patient’s treatment allocation in a secondary ‘as treated’ statistical analysis will be adjusted.

Perioperative anaesthetic management is conducted at the discretion of the attending anaesthetist. Anaesthesia is induced with IV propofol, with or without opioid and neuromuscular blockade as required. Maintenance of anaesthesia is achieved with volatile agent or propofol at the anaesthetist’s discretion. Opioids and other ancillary analgesics and antiemetics including dexamethasone can be administered intraoperatively in the usual clinical manner. IV lignocaine infusion is not permitted. Postoperative oral and/or parenteral opioids are prescribed and delivered as required according to local hospital protocols. Regional or neuraxial blockade, ancillary analgesia (including NSAIDs, cyclooxygenase (COX)−1 and COX-2 inhibitors, paracetamol, tramadol, tapentadol, and gabapentinoids) can be administered according to standard practice.

Trial research staff visit the patient daily for the first 3 days or until hospital discharge, for postoperative data collection and to liaise with the treating ward staff and acute pain service to coordinate trial solution administration with the patient care plan for each day. Follow-up data collection takes place after hospital discharge, and CPSP incidence is measured at 3 months and 12 months after surgery.

### Perioperative data collection

Preoperative data relevant to the risk of CPSP, including demographics, major co-morbidities, preoperative medications, and perioperative data, are collected. A baseline EQ-5D-3L [22] is collected in all patients for measurement of quality of life along with the Kessler K-10 Psychological Distress Scale [23], and in those who report preoperative pain in the area of the surgery, the modified Brief Pain Inventory short form (mBPI-sf) [24] and Neuropathic Pain Questionnaire (NPQ) [25] are completed.

Anaesthetic technique, medications, surgery type, time and date of surgery start and completion, and trial drug administration are documented. Time to discharge from the post-anaesthesia care unit (PACU) is recorded and quality of recovery is quantified using the QoR-15 [26] on postoperative day 1.

Acute postoperative pain severity is measured using opioid and other analgesic/antihyperalgesic drug consumption, and NRS pain scores, daily for the first 3 days or until discharge from hospital (whichever is sooner). Minimum, maximum, and mean NRS pain scores at rest and with movement or coughing are obtained each day from either the patient’s medical record or by direct questioning by trial research staff at least once daily. The Pain Catastrophising Scale (PCS), which measures catastrophic thinking related to pain [27], and the NPQ are completed on day 2 or earlier if discharged. The incidence and nature of side effects in hospital that are considered possibly related to the study drug by the treating clinicians are recorded daily. Where there was discontinuation of study drug infusion, this is recorded with the reason and time when this occurs.

The incidence of any of the trial safety endpoints are recorded. In line with National Health and Medical Research Council (NHMRC) guidelines, serious adverse events (SAE), serious adverse reactions (SAR), and suspected unexpected serious adverse reactions (SUSAR) are reported to relevant trial governance authorities and the data and safety monitoring committee (DSMC) [28].

### Post-operative follow-up at 3 and 12 months

The protocol for collection of data on CPSP is designed to meet the 6 core domain requirements set out by the recommendations of the Initiative on Methods, Measurement, and Pain Assessment in Clinical Trials (IMMPACT) [29, 30]. These are (1) pain assessment, (2) physical functioning, (3) emotional functioning, (4) participant ratings of improvement and satisfaction with treatment, (5) symptoms and adverse events, and (6) participant disposition.

**Fig. 1:**
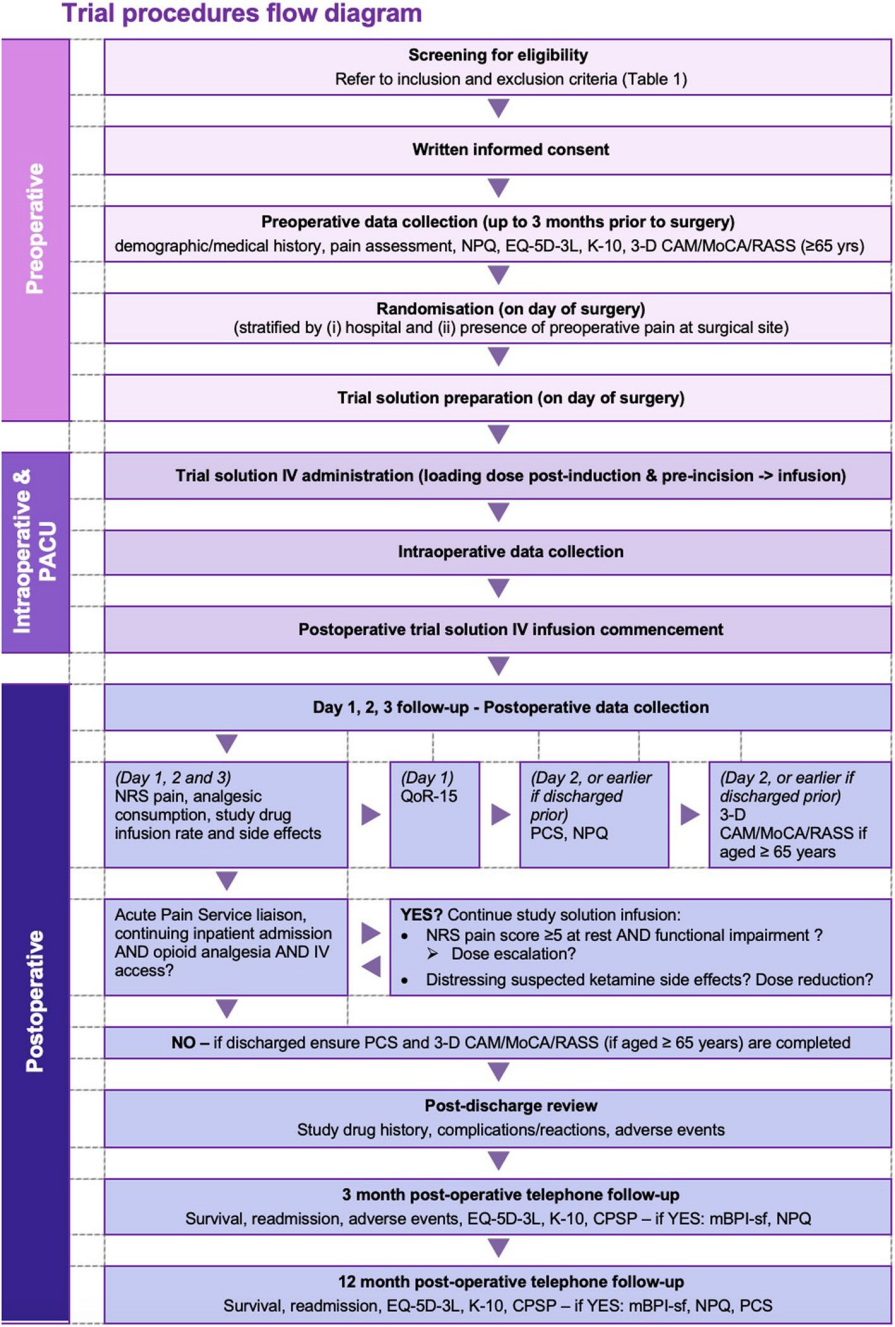
Trial process and timeline summary

Following review of the patient’s hospital medical history, research staff contact the patient by phone at 3 and 12 months after surgery. A structured interview is then conducted to determine the incidence, severity, and nature of CPSP reported by the patient. Figure 1 summarises the trial process and timeline.

### Primary endpoint: CPSP at 3 months

Patients are asked whether they have experienced pain in the area of the index surgery in the previous week. If so, details including free text description of the pain by the patient, and of any difference from preoperative pain if it was present, are recorded, and the adapted mBPI-sf and NPQ questionnaires are administered.

CPSP is defined as pain in the area of the index surgery in the previous week which either was not present preoperatively or is different in site, nature, or intensity (NRS score ≥ 2/10 difference) to any pain present at the site prior to surgery. Pain reported at interview by the patient and its recorded details are subsequently adjudicated to determine whether they meet this definition, by two members of the treatment-blinded EAC, which consists of pain medicine specialists who are all Fellows of the Facult*y* of Pain Medicine of ANZCA (ANZCA FPM). Where these two members disagree on the outcome for a given patient, further review by a third member is done as a ‘tiebreak’.

Secondary analyses will measure the incidence and treatment effect on moderate (mean NRS score ≥ 3/10 and < 5/10) and severe (mean NRS ≥ 5/10) CPSP. CPSP incidence and severity and ketamine treatment effect at 12 months will be reported as part of a subsequent long-term follow-up study.

### Secondary and exploratory endpoints

The effect of ketamine on indices of acute postoperative pain severity (opioid consumption measured as oral morphine equivalents, and NRS pain scores) on the first three postoperative days (or until hospital discharge) will be assessed. Quality of recovery in the two trial treatment arms will be compared using the QoR-15 on day 1 post-surgery.

Measures of wellbeing and psychological distress using the EQ-5D-3L and K-10 are collected in all patients prior to surgery and at 3 and 12 months post-surgery. The relationship of change from baseline to 3 and 12 months post-surgery in these variables will be examined with respect to trial treatment effect.

Patients who deny the presence of pain at follow-up are also asked if they have experienced ‘discomfort’ in the area of the index surgery in the previous week, followed by a similar pathway of questions. This pursues a secondary hypothesis that some patients may experience chronic symptoms which they may not identify as pain, particularly neuropathic symptoms, but which may adversely affect quality of life. The incidence of, and treatment effect on, this novel endpoint will be investigated as secondary trial outcomes in parallel to CPSP and reported separately.

### Database

A custom designed central electronic database has been constructed and maintained by a commercial provider (Research Path Pty Ltd, Melbourne, Victoria, Australia) for randomisation, drug inventory management, and data collection. Data entry onto the central electronic database at each participating site is done via a password-protected, online web-based portal to a secure server. The database has the facility for transmitting data queries and requests for missing data, directly or via email notifications, to site research coordinators as well as data interrogation and cross-checking algorithms to minimise incorrect data entries and maximise data capture and completeness. Data completeness and outstanding data queries are continuously updated and displayed in the database portal for the attention of the trial management team to allow minimise missing data burden. The database will be held and maintained on a secure server after the trial completes with password-protected access available through agreement with the trial steering committee’s appointed data custodian.

### Data integrity and monitoring

In accordance with Good Clinical Practice (GCP) Monitoring guidelines, all participating sites undergo routine compliance monitoring, by way of centralised and on-site monitoring of randomly selected patient records, and may include a further telephone follow-up of a randomly selected sample of patients. Consent for this by patients is included in the Participant Information and Consent Form (PICF).

### Safety endpoints and adverse event reporting and monitoring

Data on a range of adverse events among recruited patients is recorded during admission, at discharge from hospital, and at 3 months post-surgery, with complications classified according to body system (cardiovascular, respiratory, neurological, gastrointestinal, urinary system, musculoskeletal, metabolic, and haematological) and including sepsis/infection, prolongation of hospital admission, need for re-operation, hospital readmission, and death. Classification of severity follows the NHMRC Guidance on safety monitoring and reporting in clinical trials involving therapeutic goods (November 2016) [28] and Common Terminology Criteria for Adverse Events (CTCAE) [31]. An adverse event (AE) is one where the nature and severity are consistent with the current Product Information Brochure. Serious adverse events (SAEs) and suspected unexpected serious adverse reactions (SUSARs) are reported for events that are unexpected given the participant’s underlying condition, and in the opinion of the investigator, the event is possibly related to the ROCKet trial solution infusion. SAEs and SUSARs are reported to the trial coordinating centre who are responsible for informing the relevant ethics committees and the administering institution for the trial, within 24 h of the site investigators becoming aware of the event, using a dedicated reporting form. These reports are reviewed by the DSMC.

### Sample size and study power

Trial sample size was initially calculated using CPSP incidence data at 12 months post-surgery, collected from a broad sample of patients who underwent major surgery and were recruited to the ENIGMA II trial and the ROCKet pilot study [6, 7, 16]. From this data, an incidence of CPSP at 12 months post-surgery of 12% was expected in the placebo arm. A 25% reduction in the incidence of CPSP by ketamine (i.e. a decrease from 12% to 9%, a risk ratio [RR] of 0.75) was hypothesised. Using a two-sided type I error of 4.9% (applying a correction for the pre-planned interim analysis of CPSP at 12 months at *n* = 1500 that allows stopping early for superiority using a conservative Haybittle-Peto boundary) and a power of 90%, recruitment of a total of 4396 patients was planned. Incorporating a 10% lost to follow-up rate at 12 months (similar to that encountered in the ENIGMA II follow-up study) [6], this leads to a final sample size of 4884 patients or 2442 per treatment arm.

Following alignment of the timing of the trial primary endpoint with the new ICD-11 definition of CPSP in 2022 [18, 19], the sample size was not changed. The observed pooled incidence (95% CI) of CPSP at 3 months post-surgery based on blinded EAC-adjudicated CPSP data from the first 1500 recruited patients included in the planned interim analysis is 24 (22–27)%. The observed loss to follow-up rate at 3 months post-surgery is approximately 6%. Assuming the final sample size of 4884 patients is achieved and using 22% as a conservative estimate of the overall incidence of CPSP at 3 months, the study has at least 80% power to detect a clinically significant reduction of at least 15% in incidence of CPSP at 3 months by ketamine (a decrease from 23.75% to 20.25%, or RR of 0.85).

### Statistical analysis

A detailed statistical analysis plan will be developed prior to unblinding of the data collected up to 3 months after surgery. In what follows, the analysis plan using data up to and including the primary endpoint data is described. A separate plan will be written before unblinding for the data collected up to 12 months post-surgery.

The analysis will include all randomised patients, excluding patients who had their surgery cancelled post-randomisation and did not return later for their planned surgery. For the analysis of the efficacy outcomes, patients will be reported and analysed according to their randomised study arm (i.e. ‘as randomised’). For the analysis of safety outcomes, patient data will be reported and analysed according to the intervention received (i.e. ‘as treated’), whereby those who did not receive at least one study treatment will be excluded from the analysis.

The primary estimand for the ROCKet trial is defined according to the addendum to the ICH E9 on estimands and sensitivity analysis in clinical trials [32]. The trial aims to answer the specific research question: does IV ketamine, given immediately prior to and for up to 72 h following surgical incision, compared to placebo, reduce CPSP at 3 months after surgery, measured by the relative difference in the incidence/risk (i.e. RR) of CPSP at 3 months after surgery between treatment arms, regardless of the post-randomisation (intercurrent) events listed below, while assigning the patients who died to have CPSP at 3 months?

Primary estimand attributes are:

- Treatment: IV ketamine, given immediately prior to and for up to 72 h following surgical incision, compared to placebo, allocation by randomisation
- Population: consenting adult patients presenting for elective or expedited surgery under anaesthesia for a variety of major surgeries associated with CPSP
- Variable: CPSP at 3 months post-surgery
- Intercurrent events: not receiving trial solution during surgery will be handled using a treatment policy strategy, while death (expected incidence to be around 0.5% at 3 months) will be handled using a composite strategy by assigning the patients who died to have CPSP at 3 months
- Population level summary: relative difference in the incidence/risk (i.e. RR) of CPSP at 3 months after surgery between treatment arms (IV ketamine versus placebo)

The primary outcome of CPSP (yes/no) at 3 months after surgery will be analysed using a binomial log-linear regression model, with the placebo group as the reference, and in the model site, presence or absence of preoperative pain in the area of the surgery (stratification factors), and treatment. The primary hypothesis will be evaluated by obtaining the estimate of the RR for treatment and a 95.1% confidence interval (two-sided *α* = 0.049).

Binary secondary effectiveness outcomes will be analysed similar to the primary outcome and targeting a similar primary estimand, and for continuous secondary effectiveness outcomes, a linear regression model will be fitted with a model specification similar to that of the primary outcome. Sensitivity analysis will be conducted adjusting the model for pre-specified baseline covariates. Supplementary analysis targeting a different estimand with a different strategy for handling the intercurrent event of death will also be performed.

### Subgroup analyses

We plan subgroup analyses of CPSP at 3 months, including by presence or absence of preoperative pain in the area of surgery, surgical subgroup (abdominal, thoracic/breast, orthopaedic, other), sex (male, female), and age (< 65 years, ≥ 65 years). Heterogeneity of the treatment effects will be investigated by including the main subgroup effect and an interaction between the subgroup and treatment in the model. For presence of preoperative pain in the area of the surgery, we anticipate a smaller treatment effect compared to those without preoperative pain, since there is less likelihood of a preventive or pre-emptive component to any treatment effect. For the comparison of different surgical subgroups, we anticipate heterogeneity of effect. Finally, because CPSP is commoner in younger patients, patients ≥ 65 years of age are hypothesised to have a smaller treatment effect, compared to those < 65 years of age.

### Missing data

Missing values in all outcomes will be reported across treatment groups and time points. For the primary analysis of the primary and secondary efficacy outcomes, missing data will be handled using multiple imputation, assuming data are missing at random. Further analyses for the primary outcome (CPSP at 3 months post-surgery) will be conducted using (1) complete case data, assuming data are missing completely at random, and (2) the delta-adjustment method under the pattern-mixture modelling framework, assuming missingness not at random. Results will be compared with the primary analysis to investigate robustness of the findings to missing data assumptions.

### Sub-studies

Several embedded sub-studies are being conducted in parallel with the main trial in consenting recruited patients. These include a health economics analysis (in patients providing additional separate consent) using deidentified healthcare expenditure data obtained using data linkage to the national public health insurer (Medicare) and the federal government Pharmaceutical Benefits Scheme (PBS); a sub-study in recruited patients 65 years of age or over measuring ketamine treatment effect on the incidence of postoperative delirium, using a validated tool (3-D CAM administered preoperatively and on Day 2 post-surgery) [33]; and a sub-study examining pharmacokinetic and pharmacogenomic predictors of treatment effects from ketamine. Longer-term follow-up of patients reporting CPSP at 12 months is planned as a separate study funded by ANZCA, to track the natural history of the condition. These will be described in detail in later publications.

### Interim analysis

The pre-planned interim analysis by the DSMC at the availability of 12-month follow-up CPSP data from the first 1500 recruited patients was conducted in July 2022, and continuation of the trial to completion was subsequently recommended by the DSMC.

### Publication and authorship policy

Publication of trial findings will be sought in a major peer reviewed medical journal. Authorship for the primary manuscript (reporting the primary endpoint) and trial methodology paper will be coordinated by the trial writing committee and will be attributed to the trial steering committee members (chief investigator, principal investigators and trial statistician, and trial manager where not limited by journal policy on contributing author numbers) ‘on behalf of the ROCKet Trial Investigators’ (which will include the associate investigators, members of the Endpoint Adjudication Committee, trial manager and management staff, and principal investigators and research coordinators at participating centres) and the ANZCA Clinical Trials Network. Public dissemination of trial results (e.g. as a press release) is expected. Trial participants are informed that they are free to contact the chief investigator for details.

### Data sharing

No data are associated with the current article. The steering committee will oversee data sharing for the trial after it is completed. Applications by third parties to access deidentified data for secondary studies or other research purposes can be forwarded to the steering committee for consideration.

### Funding

Following pilot trial funding by ANZCA in 2014, funding for the ROCKet trial has been provided by an NHMRC Project Grant (GNT1120848, 2017), with additional funding for the Hong Kong cohort of the trial from a grant from the Hong Kong Research Grants Council (14112718). Following the trial recruitment delays arising from the SARS CoV-2 pandemic, and recommendation from the trial DSMC after the planned interim analysis to continue to recruit to the original target, additional funding was obtained from an NHMRC Clinical Trials and Cohort Studies Grant (GNT2023989, 2022) to achieve this.

## Trial status

Trial recruitment commenced on 19 December 2017. In total, 36 hospitals have recruited patients to the trial across Australia, New Zealand, and Hong Kong. The current protocol version is v5.0, 7 July 2023. The trial is expected to complete recruitment in the first half of 2025.

### Progress and COVID-19 impact

In common with many perioperative clinical trials, recruitment has been heavily impacted by the SARS CoV-2 pandemic, which led to severe reductions in elective surgical throughput in public hospitals, along with research staff redeployment and furloughing, over a 2-year period from March 2020. Trial recruitment has not returned to its pre-pandemic peak levels. This has led to significant delays in recruitment and funding shortfall. Trial completion has been made possible by an additional grant from the NHMRC.

## Discussion

The importance of CPSP as a healthcare outcome arises not only from its frequency as a complication of surgery but also from its impact on quality of life, which can be measured using quality-adjusted life years (QALYs) and associated healthcare costs. The economic costs of chronic pain have been studied in several countries [34–36]. Using data from the United States on costs of neuropathic pain, Schaefer et al. [35] estimate these costs are USD$25,000 per year per patient. In Australia, the cost of chronic pain in the community (loss of productivity and direct medical costs) was estimated at AUD$22,588 per year per patient in 2018 with pain severity profiles very similar to those measured by us for CPSP encountered in the ENIGMA trials [37–39]. CPSP contributes significantly to the burden of chronic pain in the community [37]. This is deeply concerning, since there is only limited evidence for the effectiveness of any intervention to treat established chronic pain. Furthermore, funding and availability of chronic pain services are seriously inadequate to meet demand, with long waiting times [40, 41].

Studies into treatments aimed at preventing progression of acute postoperative pain to CPSP have produced mixed, but generally disappointing, results. The relatively small number of studies that have looked at CPSP have been underpowered and are characterised by heterogenous methodologies [42]. Mechanistic issues such as the timing of drug administration (‘pre-emptive’ versus ‘preventive’ strategies) add to this uncertainty [43–45]. Metanalysis of small trial data on potential therapies has suffered from significant heterogeneity as well as susceptibility to type 1 and 2 statistical error and risk of publication bias [46, 47].

For these reasons, identifying effective strategies for primary prevention of CPSP from adequately powered prospective trials is of paramount importance. The ROCKet trial is pursuing one of the most promising available treatments, IV ketamine, for this purpose. The trial protocol is deliberately pragmatic for two reasons, shared with most large multicentre effectiveness trials. Firstly, successful delivery of the intervention across a wide collaboration of participating hospitals and clinicians requires that the intervention be practical and adaptable to routine clinical practice. The sub-anaesthetic dose regimen chosen sits within the range studied in the majority of previous smaller trials on ketamine effect on acute pain reviewed in 2018 American consensus guidelines by expert societies [48] and is commonly employed in clinical practice as part of a multi-modal analgesic strategy for perioperative pain management, where its psychomimetic side effects, such as hallucinations, dreaming, and dysphoria, are less severe [49]. Secondly, the flexibility in postoperative infusion rates embedded in the trial protocol, in response to refractory acute pain or suspected side effects, enhances the practicality of the treatment regimen, reflecting standard clinical management [49].

A further pragmatic consideration is the permissible variation in duration of ketamine infusion. While seeking to maximise this or mandating a minimum duration of three postoperative days might be considered desirable on a mechanistic basis, such a trial protocol would be inconsistent with many perioperative management protocols informed by ERAS (Enhanced Recovery After Surgery) principles which are now a widespread standard of care and would heavily curtail compliance with the trial protocol, particularly in view of the diverse mix of surgery included in the trial. Consequently, the results of the pragmatic trial design are more likely to be generalisable and find ready translation to routine practice. Clinicians can have more confidence in the robustness of the findings of large effectiveness trials in reliably informing changes to clinical practice to improve patient outcomes, than is the case with synthesis of data from small single centre efficacy trials [46, 47, 50, 51].

A limitation of large multicentre trial design, on the other hand, particularly where longer-term clinical outcomes such as CPSP are being studied, is the need for a pragmatic model of remote data collection. An example of this in the ROCKet trial is the need to identify neuropathic pain using tools that do not include face to face clinical examination, for example to test for the presence of allodynia. The NPQ was designed for this purpose and was chosen over alternatives such as the DN4 (Doleur Neuropathique 4) questionnaire for this reason [25, 52]. While potentially having less precision in identification of neuropathic pain characteristics, associations of neuropathic pain with other trial endpoints are expected to be robust given the large trial sample size.

Because they collect large amounts of phenotypic data, large randomised controlled trials provide unique but often relatively brief opportunities for prospective exploratory and mechanistic substudies which help inform interpretation of trial findings and directions for future research and precision medicine. This aspect of large trial impact is often overlooked in the funding process, which can easily result in lost opportunities to value add to the final contribution made by a large trial. An example of this is the collection of a large amount of prospective data on ketamine effect on measures of acute postoperative pain severity. The evidence base for the widespread use of perioperative ketamine for acute postoperative pain management is very heterogeneous, with wide variation in practice and study populations among numerous small, single-centre trials and little data to reliably inform a potential dose–response relationship [11]. The ROCKet trial is expected to substantially improve this in addition to its primary aim to address a potential effect on CPSP.

Regardless of the primary finding of the trial on the effectiveness of ketamine in reducing CPSP, the ROCKet trial will provide a wealth of high-quality, prospective data on the epidemiology and natural history of CPSP which will help drive further research into prevention and management of the condition.

## Data Availability

No data are associated with this article.

## Abbreviations

ANZCA, CTN: Australian and New Zealand College of Anaesthetists
CPSP: Chronic post-surgical pain
COX: Cyclooxygenase
CTN: Clinical Trials Network
DSMC: Data and safety monitoring committee
DN4: Doleur Neuropathique 4
ENIGMA: Evaluation of Nitrous oxide In the Gas Mixture in Anaesthesia
FPM: Facult*y* of Pain Medicine
IASP: International Association for the Study of Pain
IMMPACT: Methods, Measurement, and Pain Assessment in Clinical Trials
IV: Intravenous
K-10: Kessler K-10 Psychological Distress Scale
mBPI-sf: Modified Brief Pain Inventory short form
NHMRC: National Health and Medical Research Council
NMDA: N-Methyl-D-aspartate
NPQ: Neuropathic Pain Questionnaire
NRS: Numerical Rating Scale
NSAIDs: Non-steroidal anti-inflammatory drugs
PACU: Post-anaesthesia care unit
PBS: Pharmaceutical Benefits Scheme
PCS: Pain Catastrophising Scale
PICF: Participant Information and Consent Form
QALYs: Quality-adjusted life years
ROCKet: *Re*duction *O*f *C*hronic post-surgical pain with *Ket*amine
SAE: Serious adverse events
SAR: Serious adverse reactions
SUSAR: Suspected unexpected serious adverse reactions
SPIRIT: Standard Protocol Items Recommendations for Interventional Trials
3-D CAM: 3-D Confusion Assessment Method

## References

[1] Macrae WA. Chronic pain after surgery. Br J Anaesth. 2001;87:88–98. 11460816 10.1093/bja/87.1.88

[2] Wicksell R, Olsson G. Predicting and preventing chronic postsurgical pain and disability. Anesthesiology. 2010;113:1260–1. 20966742 10.1097/ALN.0b013e3181da89f8

[3] Katz J, Seltzer Z. Transition from acute to chronic postsurgical pain: risk factors and protective factors. Expert Rev Neurother. 2009;9(5):723–44. 19402781 10.1586/ern.09.20

[4] Hinrichs-Rocker A, Schulz K, Järvinen I, Lefering R, Simanski C, Neugebauer EA. Psychosocial predictors and correlates for chronic post-surgical pain (CPSP) – a systematic review. Eur J Pain. 2009;13:719–30. 18952472 10.1016/j.ejpain.2008.07.015

[5] Johansen A, Romundstad L, Nielsen CS, Schirmer H, Stubhaug A. Chronic postsurgical pain in a general population: prevalence and predictors in the Tromso study. Pain. 2012;153(7):1390–6. 22445291 10.1016/j.pain.2012.02.018

[6] Chan MT, Peyton PJ, Myles PS, et al. Chronic postsurgical pain in the Evaluation of Nitrous Oxide in the Gas Mixture for Anaesthesia (ENIGMA)-II trial. Br J Anaesth. 2016;117(6):801–11. 27956679 10.1093/bja/aew338

[7] Chan M, Wan A, Gin T, Leslie K, Myles P. Chronic postsurgical pain after nitrous oxide anesthesia. Pain. 2011;152(11):2514–20. 21889262 10.1016/j.pain.2011.07.015

[8] Treede RD, Rief W, Barke A, Aziz Q, Bennett MI, Benoliel R, Cohen M, Evers S, Finnerup NB, First MB, Giamberardino MA, Kaasa S, Korwisi B, Kosek E, Lavand’homme P, Nicholas M, Perrot S, Scholz J, Schug S, Smith BH, Svensson P, Vlaeyen JWS, Wang SJ. Chronic pain as a symptom or a disease: the IASP Classification of Chronic Pain for the International Classification of Diseases (ICD-11). Pain. 2019;160(1):19–27. 30586067 10.1097/j.pain.0000000000001384

[9] Schug SA, Lavand’homme P, Barke A, et al. The IASP Taskforce for the Classification of Chronic Pain. The IASP classification of chronic pain for ICD-11: chronic postsurgical or posttraumatic pain. Pain. 2019;160:45–52. 30586070 10.1097/j.pain.0000000000001413

[10] Herrero JF, Laird JM, López-García JA. Wind-up of spinal cord neurones and pain sensation: much ado about something? Prog Neurobiol. 2000;61(2):169–203. 10704997 10.1016/s0301-0082(99)00051-9

[11] Aguiar P, Sousa M, Lima D. NMDA channels together with L-type calcium currents and calcium-activated nonspecific cationic currents are sufficient to generate windup in WDR neurons. J Neurophysiol. 2010;104:1155–66. 20554833 10.1152/jn.00834.2009

[12] McCartney CJL, Sinha A, Katz J. A qualitative systematic review of the role of N-methyl-D-aspartate receptor antagonists in preventive analgesia. Anesth Analg. 2004;98:1385–400. 15105220 10.1213/01.ane.0000108501.57073.38

[13] Laskowski K, Stirling A, McKay WP, Lim HJ. A systematic review of intravenous ketamine for postoperative analgesia. Can J Anaesth. 2011;58(10):911–23. 21773855 10.1007/s12630-011-9560-0

[14] Chaparro L, Smith S, Moore R, Wiffen P, Gilron I. Pharmacotherapy for the prevention of chronic pain after surgery in adults. Cochrane Database Syst Rev. 2013;2013(7):CD008307. 10.1002/14651858.CD008307.pub2. 10.1002/14651858.CD008307.pub2 PMC6481826 23881791

[15] Carley ME, Chaparro LE, Choini M, et al. Pharmacotherapy for the prevention of chronic pain after surgery in adults: an updated systematic review and meta-analysis. Anesthesiology. 2021;135:304–25. 34237128 10.1097/ALN.0000000000003837

[16] Boney O, Bell M, Bell N, et al. Identifying research priorities in anaesthesia and perioperative care: final report of the joint National Institute of Academic Anaesthesia/James Lind Alliance Research Priority Setting Partnership. BMJ Open. 2015Dec 16;5(12):e010006. 26674506 10.1136/bmjopen-2015-010006 PMC4691782

[17] Chan A-W, Tetzlaff JM, Gøtzsche PC, Altman DG, Mann H, Berlin J, Dickersin K, Hróbjartsson A, Schulz KF, Parulekar WR, Krleža-Jerić K, Laupacis A, Moher D. SPIRIT 2013 Explanation and Elaboration: guidance for protocols of clinical trials. BMJ. 2013;346: e7586. 23303884 10.1136/bmj.e7586 PMC3541470

[18] Peyton P, Wu C, Jacobson T, Hogg M, Zia F, Leslie K. The effect of a perioperative ketamine infusion on the incidence of chronic post-surgical pain – a pilot study. Anaesth Intens Care. 2017;45(4):459–65. 10.1177/0310057X1704500408 28673215

[19] Myles PS, Corcoran TB, Chan MTV, Asghari-Jafarabadi M, Wu WKK, Peyton P, Leslie K, Forbes A. Dexamethasone and chronic postsurgical pain: a propensity score-matched analysis of a large trial. Br J Anaesth. 2024;133:103–10. 38267338 10.1016/j.bja.2023.12.031

[20] Lee M, Silverman S, Hanse H, et al. A comprehensive review of opioid-induced hyperalgesia. Pain Physician. 2011;14:145–61. 21412369

[21] Koppert W, Sittl R, Scheube K, et al. Differential modulation of remifentanil-induced analgesia and postinfusion hyperalgesia by S-ketamine and clonidine in humans. Anesthesiology. 2003;99:152–9. 12826855 10.1097/00000542-200307000-00025

[22] EuroQolResearchFoundation. EQ-5D-5L UserGuide, 2019. Available from: https://euroqol.org/publications/user-guides.

[23] Kessler RC, Barker PR, Colpe LJ, et al. Screening for serious mental illness in the general population. Arch Gen Psychiatry. 2003;60(2):184–9. 12578436 10.1001/archpsyc.60.2.184

[24] Mendoza T, Mayne T, Rublee D, Cleeland C. Reliability and validity of a modified Brief Pain Inventory short form in patients with osteoarthritis. Eur J Pain. 2006;10(4):353–61. 16051509 10.1016/j.ejpain.2005.06.002

[25] Krause S, Backonja MM. Development of a Neuropathic Pain Questionnaire. Clin J Pain. 2003;19:306–14. 12966256 10.1097/00002508-200309000-00004

[26] Stark PA, Myles PS, Burke JA. Development and psychometric evaluation of a postoperative quality of recovery score: the QoR-15. Anesthesiology. 2013;118(6):1332–40. 23411725 10.1097/ALN.0b013e318289b84b

[27] Sullivan MJL, Bishop SR, Pivik J. The Pain Catastrophizing Scale: development and validation. Psychol Assess. 1995;7:524–32.

[28] NHMRC Guidance: safety monitoring and reporting in clinical trials involving therapeutic goods. Canberra: National Health and Medical Research Council, 2016.

[29] Dworkin RH, Turk DC, Farrar JT, et al. Core outcome measures for chronic pain clinical trials: IMMPACT recommendations. Pain. 2005;113:9–19. 15621359 10.1016/j.pain.2004.09.012

[30] Gewandter JS, Dworkin RH, Turk DC, et al. Research design considerations for chronic pain prevention clinical trials: IMMPACT recommendations. Pain. 2015;156(7):1184–97. 25887465 10.1097/j.pain.0000000000000191 PMC5769693

[31] Common Terminology Criteria for Adverse Events (CTCAE). Version 4.0 Published: May 28, 2009 (v4.03: June 14, 2010). U.S. Department Of Health And Human Services; National Institutes of Health; National Cancer Institute.

[32] https://database.ich.org/sites/default/files/E9-R1_Step4_Guideline_2019_1203.pdf. Accessed 25th April 2024.

[33] Hudetz J, Patterson K, Iqbal Z, et al. Ketamine attenuates delirium after cardiac surgery with cardiopulmonary bypass. J Cardiothorac Vasc Anesth. 2009;23(5):651–7. 19231245 10.1053/j.jvca.2008.12.021

[34] Ekman M, Jönhagen S, Hunsche E, Jönsson L*.* Burden of illness of chronic low back pain in Sweden: a cross-sectional, retrospective study in primary care setting. Spine (Phila Pa 1976). 2005;30(15):1777–85. 10.1097/01.brs.0000171911.99348.90 16094281

[35] Schaefer C, Sadosky A, Mann R, et al. Pain severity and the economic burden of neuropathic pain in the United States: BEAT Neuropathic Pain Observational Study. Clinicoecon Outcomes Res. 2014;6:483–96. 25378940 10.2147/CEOR.S63323 PMC4218900

[36] Pérez C, Navarro A, Saldaña MT, Wilson K, Rejas J. Modelling the predictive value of pain intensity on costs and resources utilization in patients with peripheral neuropathic pain. Clin J Pain. 2015;31(3):273–9. 24762867 10.1097/AJP.0000000000000110

[37] Access Economics & MBF Foundation. The high price of pain: the economic impact of chronic pain in Australia – Pain Management Research Institute, University of Sydney. 2007; p13–14.

[38] Deloitte Access Economics/Painaustralia. The cost of pain in Australia. March 2019.

[39] Blyth FM, March LM, Cousins MJ. Chronic pain-related disability and use of analgesia and health services in a Sydney community. Med J Aust. 2003;179:84–7. 12864718 10.5694/j.1326-5377.2003.tb05441.x

[40] Semple TJ, Hogg MN. Waiting in pain. MJA. 2012;196(6):372–3. 22471528 10.5694/mja12.10148

[41] Cousins MJ. Unrelieved pain: a major health care priority. MJA. 2012;196(6):373–4. 22471529 10.5694/mja12.10181

[42] Clarke H, Bonin RP, Orser BA, Englesakis M, Wijeysundera DN, Katz J. The prevention of chronic postsurgical pain using gabapentin and pregabalin: a combined systematic review and meta-analysis. Anesth Analg. 2012;115(2):428–42. 22415535 10.1213/ANE.0b013e318249d36e

[43] Møiniche S, Kehlet H, Dahl JB. A qualitative and quantitative systematic review of preemptive analgesia for postoperative pain relief the role of timing of analgesia. Anesthesiology. 2002;96:725–41. 11873051 10.1097/00000542-200203000-00032

[44] Kissin I. A call to reassess the clinical value of preventive (pre-emptive) analgesia. Anesth Analg. 2011;113(5):977–8. 22021795 10.1213/ANE.0b013e31822d39f9

[45] Katz J, Clarke H, Seltzer Z. Preventive analgesia: quo vadimus? Anesth Analg. 2011;113:1242–53. 21965352 10.1213/ANE.0b013e31822c9a59

[46] Sivakumar H, Peyton PJ. Poor agreement in significant findings between meta-analyses and subsequent large randomised trials in peri-operative medicine. Br J Anaesth. 2016;117:431–41. 28077529 10.1093/bja/aew170

[47] Chong SW, Collins NF, Wu C, Liskaser G, Peyton PJ. The relationship of study findings to publication outcome in anesthesia research: a retrospective observational study examining publication bias. Can J Anesth. 2016;63:682–90. 27038290 10.1007/s12630-016-0631-0

[48] Schwenk ES, Viscusi ER, Buvanendran A, Hurley RW, Wasan AD, Narouze S, Bhatia A, Davis FN, Hooten WM, Cohen SP. Consensus guidelines on the use of intravenous ketamine infusions for acute pain management from the American Society of Regional Anesthesia and Pain Medicine, the American Academy of Pain Medicine, and the American Society of Anesthesiologists. Reg Anesth Pain Med. 2018;43:456–66. 29870457 10.1097/AAP.0000000000000806 PMC6023582

[49] Lloyd-Donald PJ, Peyton PJ. Survey of administration of intravenous ketamine for perioperative pain management in Australia and New Zealand. In press, Anaesth Intensive Care, Accepted for publication 13th July 2024. 10.1177/0310057X241309655 39939123

[50] Yusuf S, Collins R, Peto R. Why do we need some large simple randomised trials? Stat Med. 1994;3:409–20. 10.1002/sim.4780030421 6528136

[51] Myles PS. Why we need large randomised studies in anaesthesia. Br J Anaesth. 1999;83:833–4. 10700777 10.1093/bja/83.6.833

[52] Bouhassira D, Attal N, Alchaar H, et al. Comparison of pain syndromes associated with nervous or somatic lesions and development of a new neuropathic pain diagnostic questionnaire (DN4). Pain. 2005;114:29–36. 15733628 10.1016/j.pain.2004.12.010

